# Methanogenic Biodegradation of *iso*-Alkanes by Indigenous Microbes from Two Different Oil Sands Tailings Ponds

**DOI:** 10.3390/microorganisms9081569

**Published:** 2021-07-23

**Authors:** Mohd Faidz Mohamad Shahimin, Julia M. Foght, Tariq Siddique

**Affiliations:** 1 Department of Renewable Resources, University of Alberta, Edmonton, AB T6G 2G7, Canada; faidz.shahimin@gmail.com; 2 Department of Biological Sciences, University of Alberta, Edmonton, AB T6G 2E9, Canada; Julia.foght@ualberta.ca

**Keywords:** methanogenesis, oil sands tailings, *iso*-alkanes, preferential hydrocarbon biodegradation, *Peptococcaceae*

## Abstract

*iso*-Alkanes, a major fraction of the solvents used in bitumen extraction from oil sand ores, are slow to biodegrade in anaerobic tailings ponds. We investigated methanogenic biodegradation of *iso*-alkane mixtures comprising either three (2-methylbutane, 2-methylpentane, 3-methylpentane) or five (2-methylbutane, 2-methylpentane, 2-methylhexane, 2-methylheptane, 2-methyloctane) *iso*-alkanes representing paraffinic and naphtha solvents, respectively. Mature fine tailings (MFT) collected from two tailings ponds, having different residual solvents (paraffinic solvent in Canadian Natural Upgrading Limited (CNUL) and naphtha in Canadian Natural Resources Limited (CNRL)), were amended separately with the two mixtures and incubated in microcosms for ~1600 d. The indigenous microbes in CNUL MFT produced methane from the three-*iso*-alkane mixture after a lag of ~200 d, completely depleting 2-methylpentane while partially depleting 2-methylbutane and 3-methylpentane. CNRL MFT exhibited a similar degradation pattern for the three *iso*-alkanes after a lag phase of ~700 d, but required 1200 d before beginning to produce methane from the five-*iso*-alkane mixture, preferentially depleting components in the order of decreasing carbon chain length. *Peptococcaceae* members were key *iso*-alkane-degraders in both CNUL and CNRL MFT but were associated with different archaeal partners. Co-dominance of acetoclastic (*Methanosaeta*) and hydrogenotrophic (*Methanolinea* and *Methanoregula*) methanogens was observed in CNUL MFT during biodegradation of three-*iso*-alkanes whereas CNRL MFT was enriched in *Methanoregula* during biodegradation of three-*iso*-alkanes and in *Methanosaeta* with five-*iso*-alkanes. This study highlights the different responses of indigenous methanogenic microbial communities in different oil sands tailings ponds to *iso*-alkanes.

## 1. Introduction

Alkanes are typically a major component of crude oils and petroleum products, and are among the least chemically reactive organic compounds [1]. Despite the inert nature of alkanes, their utilization by microbes as sole carbon and energy sources under aerobic conditions has been well-known for a century [2]. In contrast, microbial metabolism of alkanes under anaerobic conditions was convincingly demonstrated only in the last three decades [3,4,5,6,7], and primarily has focused on *n*-alkanes. Reports of anaerobic biodegradation of *iso*- and cycloalkanes are still scarce due to their relative recalcitrance compared to *n*-alkanes [8,9,10,11]. Biodegradation of *iso*- and cycloalkanes is much slower under anaerobic conditions than aerobic conditions where degradation occurs within a few days of incubation [12,13,14]. Nonetheless, studies of the biodegradability of *iso*- and cycloalkanes under anaerobic conditions are important since these isomers are also significant oil components that can impact the environment.

Tailings ponds are essential land features of surface-mined oil sands operations in northern Alberta, Canada and are a source of greenhouse gas (GHG) emissions, particularly methane (CH_4_) [15]. Unrecovered extraction solvents, of which alkanes are a major constituent, represent a small component of the oil sands processing effluents comprising water, sand, silt, clay, and unextracted bitumen that are deposited into enormous oil sands tailings ponds where they sustain methanogenesis [16]. Different operators use extraction solvents having different compositions [17] ranging from paraffinic (almost entirely aliphatic C_5_ and C_6_ hydrocarbons), as used by Canadian Natural Upgrading Limited (CNUL; formerly known as Shell Albian Sands Inc.), to various naphtha distillates (a mixture of mostly C_5_–C_10_ aliphatics plus monoaromatics) such as the light naphtha used by Canadian Natural Resources Limited (CNRL). These different residual solvent inputs are reflected in development of microbial communities unique to each tailings pond [18,19].

Previous studies [20,21,22] revealed that *n*-alkanes were important substrates for the indigenous methanogenic microbial community in mature fine tailings (MFT) [23] retrieved from the Syncrude Canada Limited tailings pond called Mildred Settling Lake Basin, whereas other major components such as *iso*- and cycloalkanes were degraded only after prolonged incubation in the laboratory [8,9]. Furthermore, we found that MFT microbes preferentially degraded *n*-alkanes present in their own tailings solvent versus those prevalent in solvents from other tailings ponds [11,20,21,22,24,25]. The objective of the current study was to determine whether this pattern of preference carried over to recalcitrant ‘secondary substrates’ such as *iso*-alkanes in these two tailings ponds, which differ in age, residual solvent type, and physicochemical properties [25].

Using solvent composition information, we prepared two mixtures of *iso*-alkanes to reflect the composition of solvents used in CNUL and CNRL operations. The light paraffinic CNUL solvent was represented by a mixture of three *iso*-alkanes (2-methylbutane, 2-methylpentane, and 3-methylpentane), the major *iso*-alkanes in the CNUL solvent [11]. The CNRL naphtha solvent was represented by a mixture of five *iso*-alkanes (2-methylbutane, 2-methylpentane, 2-methylhexane, 2-methylheptane, and 2-methyloctane) present in the CNRL solvent [25]. We incubated each MFT with its cognate solvent and with the other ‘exotic’ solvent in sealed microcosms, measuring CH_4_ production, the extent and patterns of biodegradation of individual *iso*-alkanes within the mixtures, and changes to the microbial communities exposed to the *iso*-alkane mixtures, as indicators of biodegradation potential.

Since *iso*-alkanes are significant components of all extraction solvents used by surface-mined oil sands operators, evaluating their biodegradation by MFT from different tailings ponds provides insights into anaerobic *iso*-alkane biodegradation processes. The information improves our understanding of methanogenesis in tailings ponds, which not only generates GHG but also drastically affects the management and reclamation of oil sands tailings under wet reclamation scenarios such as establishment of end-pit lakes, though accelerated consolidation of tailings [26,27]. Additionally, findings from this study will further improve existing models of GHG emissions from tailings ponds [15,28] by providing additional data to refine kinetics parameters used in the models.

## 2. Materials and Methods

### 2.1. Chemicals and Materials

High-purity *iso*-alkanes were purchased as follows: 2-methylbutane (2-MC_4_; >99%; CAS#78-78-4) and 2-methylpentane (2-MC_5_; >99%; CAS#107-83-5) from Alfa Aesar, Haverhill, MA, USA; 2-methylhexane (2-MC_6_: >99%; CAS#591-76-4) and 2-methylheptane (2-MC_7_: >99%; CAS#592-27-8) from Acros Organics, Fair Lawn, NJ, USA; 2-methyloctane (2-MC_8_: >99%; CAS#34464-40-9) from MP Biomedicals, Irvine, CA, USA; and 3-methylpentane (3-MC_5_: >99%; CAS#96-14-0) from Sigma-Aldrich, Saint Louis, MO, USA. Two mixtures of these *iso*-alkanes were prepared separately in sealed vials: the mixture of three *iso*-alkanes (“M-3I”) comprised 2-methylbutane, 2-methylpentane, and 3-methylpentane, and the mixture of five *iso*-alkanes (“M-5I”) comprised 2-methylbutane, 2-methylpentane, 2-methylhexane, 2-methylheptane, and 2-methyloctane.

Methanogenic CNUL and CNRL MFT were collected from Muskeg River Mine Tailings Pond and Horizon Tailings Ponds by the respective oil sands operators. Details of geographical coordinates and physical and chemical properties of the MFT samples have been described previously [21].

### 2.2. Preparation of Microcosms

All microcosms (158 mL serum bottles) were prepared under methanogenic conditions as described previously [21]. Briefly, 50 mL methanogenic medium [29] was added into sterile microcosms containing 50 mL of CNUL or CNRL MFT, sealed, and the headspace was purged with 30% CO_2_, balance N_2_. The microcosms were incubated stationary in the dark at ~20 °C for at least two weeks to deplete indigenous substrates and acclimate the cultures to the incubation conditions. Immediately before amending with the *iso*-alkane mixtures, the headspace was flushed with 30% CO_2_, balance N_2_ to remove any background CH_4_. The prepared hydrocarbon mixtures were then added to each microcosm via syringe immediately after the headspace was purged, providing 20–70 mg per 100 mL of culture for each compound; the mass of each *iso*-alkane added at day 0 is shown in Table 1. Triplicate heat-killed controls (autoclaved for 60 min on each of four consecutive days) were amended with the same mixtures at the same concentrations. Duplicate unamended live microcosms were also prepared as viable baseline controls to account for metabolism of any residual endogenous substrates in the MFT that could result in CH_4_ production. All microcosms were incubated stationary at ~20 °C in the dark. Periodic analyses of CH_4_ and hydrocarbons were performed as described below to monitor degradation of *iso*-alkanes over time.

### 2.3. Chemical Analyses

CH_4_ production was measured in all microcosms by removing 50 μL of headspace periodically from each microcosm via syringe for analysis by gas chromatography with a flame ionization detector (GC-FID) as previously described [30]. CH_4_ in unamended and abiotic controls cultures was concurrently measured to take into account biodegradation of endogenous organic compounds in unamended cultures and to confirm that there was no biological generation of CH_4_ in abiotic controls.

At intervals, the concentration of *iso*-alkanes in all microcosms was determined by aseptically removing 1 mL of culture and extracting hydrocarbons using 10 mL methanol (Fisher Scientific) in a sealed 20 mL EPA glass vial. The vials were mechanically shaken for 30 min at room temperature then particles in the vials were allowed to settle for 30 min. One milliliter of the supernatant was transferred to a 44 mL EPA glass vial, filled to the top with ultrapure water to avoid any headspace, and capped. The vials were sonicated for 10 min prior to analysis using purge and trap GC-FID as detailed previously [20].

### 2.4. Stoichiometry of iso-Alkane Mineralization under Methanogenic Conditions

Complete methanogenic biodegradation of *iso*-alkanes will result in the production of CH_4_ and CO_2_ as final products. The modified Symons and Buswell simple stoichiometric equation [31] was used to calculate the theoretical maximum CH_4_ and CO_2_ production, assuming complete mineralization of *iso*-alkanes to CH_4_ and CO_2_ without any incorporation of the carbon into biomass. The calculated theoretical CH_4_ was then compared to the measured CH_4_. The developed equations are as follows

2-methylbutane (2-MC_4_)     C_5_H_12_ + 2H_2_O → CO_2_ + 4CH_4_ (1)

2-methylpentane (2-MC_5_)     C_6_H_14_ + 2.5H_2_O → 1.25CO_2_ + 4.75CH_4_ (2)

3-methylpentane (3-MC_5_)     C_6_H_14_ + 2.5H_2_O → 1.25CO_2_ + 4.75CH_4_ (3)

2-methylhexane (2-MC_6_)     C_7_H_16_ + 3H_2_O → 1.5CO_2_ + 5.5CH_4_ (4)

2-methylheptane (2-MC_7_)     C_8_H_18_ + 3.5H_2_O → 1.75CO_2_ + 6.25CH_4_ (5)

2-methyloctane (2-MC_8_)     C_9_H_20_ + 4H_2_O → 2CO_2_ + 7CH_4_ (6)

### 2.5. Nucleic Acid Extraction and Purification

DNA extraction and purification were performed at day 0 (initial microbial community) and thereafter at intervals corresponding to phases of significant methane production, using the method previously described [21]. Briefly, DNA was extracted from triplicate 300-μL samples in screw-cap microcentrifuge tubes (Fisher Scientific) containing 1 g of 2.5 mm and 0.1 mm zirconia silica beads (1:1 *w*/*w*), 100 mM NaH_2_PO_4_ (pH 8.0), 100 mM NaCl, 500 mM Tris pH 8.0, 10% sodium dodecyl sulfate, and chloroform–isoamyl alcohol (24:1), and processed in a PowerLyzer™ 24 Bench Top Bead-based Homogenizer (MO BIO Laboratories Inc., Carlsbad, CA, USA) at 3400 rpm for 45 s. The samples were spun at full speed (15,000 rpm) in a microfuge for 5 min to sediment the debris and the supernatant was collected for subsequent DNA recovery. The extraction procedure was repeated on each sample, without pooling the supernatants. For each supernatant, 7 M NH_4_CH_3_CO_2_ was added to 2.5 M final concentration, mixed gently and microcentrifuged to precipitate proteins. Supernatants were transferred to fresh tubes plus 0.6 volumes of isopropanol and incubated overnight at 4 °C. DNA was precipitated by microfuge at full speed for 30 min. After decanting the isopropanol, the DNA pellet was air-dried for 60 min and dissolved in 50 µL nuclease-free water (Integrated DNA Technologies). The DNA from both extractions of each sample was then pooled. The pooled DNA was checked for purity and concentration using a Nanodrop-1000 Spectrophotometer before subsequent PCR amplification.

### 2.6. PCR Amplification and Bioinformatics

All purified DNA samples were subjected to polymerase chain reaction (PCR) amplification using the primer set 454T-RA/454T-FB targeting the V6-V8 regions of the 16S rRNA gene universal for Bacteria and Archaea [32,33,34]. The PCR reaction and amplification and the subsequent DNA purification were performed following methods published previously [21], and negative controls containing only PCR reagents and nuclease-free water were processed in parallel for quality control. The 16S rRNA amplicons were pyrosequenced using GS FLX Titanium Series Kit XLR70 (Roche) at McGill University Génome Québec Innovation Centre, Canada. The raw pyrosequencing data were analyzed using the Phoenix 2.0 pipeline [35]. Taxonomic annotation was performed on quality-controlled reads, using the SILVA database with average neighbor clustering at 5% maximum distance cut-off to define operational taxonomic units (OTUs). As observed in our previous studies, most archaeal sequences from MFT were confidently identified to species level, whereas bacterial OTUs often could be affiliated only to family- or order-level at best. All raw pyrosequences (~1700–15,000 reads per sample) have been submitted to NCBI Sequence Read Archive under SRA number SRP052814.

## 3. Results

### 3.1. Methanogenic Biodegradation of iso-Alkanes

**Figure 1:**
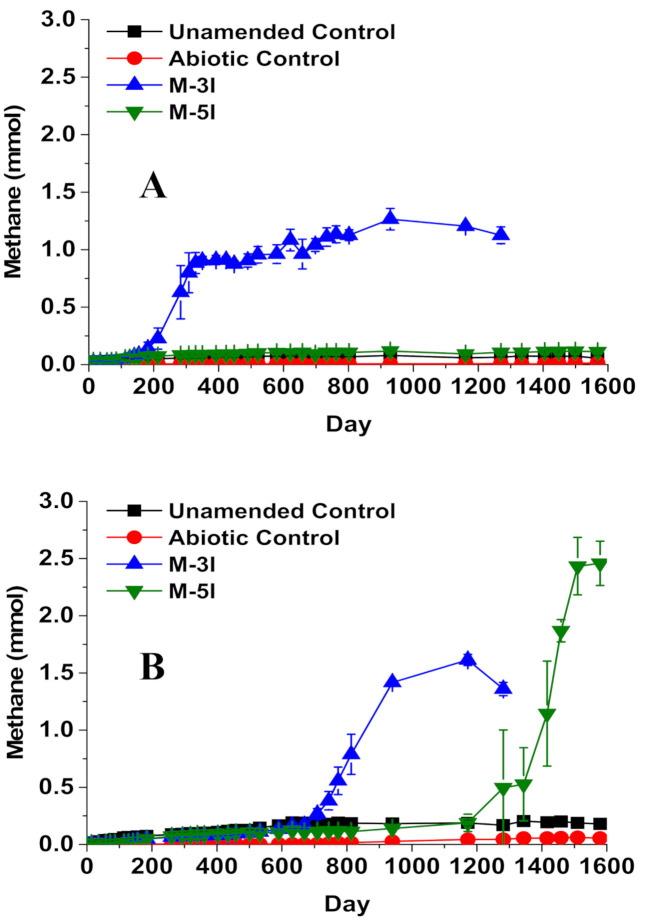
Cumulative methane production in microcosms containing either (**A**) CNUL MFT or (**B**) CNRL MFT, amended with *iso*-alkane mixtures and incubated under methanogenic conditions for ~1600 d. The three-*iso*-alkane mixture (M-3I) comprised 2-methylbutane (2-MC_4_), 2-methylpentane (2-MC_5_), and 3-methylpentane (3-MC_5_), whereas five-*iso*-alkane mixture (M-5I) comprised 2-MC_4_, 2-MC_5_, 2-methylhexane (2-MC_6_), 2-methylheptane (2-MC_7_), and 2-methyloctane (2-MC_8_). Symbols represent the mean value of measurements from triplicate microcosms and error bars, where visible, represent one standard deviation.

Microcosms containing MFT were amended with one of two *iso*-alkane mixtures, incubated for up to ~1600 d, and periodically monitored for CH_4_ production and hydrocarbon biodegradation. Neither the heat-killed amended microcosms (abiotic control) nor the unamended microcosms (baseline control) containing either CNUL or CNRL MFT produced significant CH_4_ (<0.2 mmol; Figure 1), indicating that any native substrates remaining in the MFT did not contribute substantially to CH_4_ production during incubation.

CNUL MFT amended with the three-*iso*-alkane mixture (M-3I) exhibited a lag phase of ~110 d before cumulative CH_4_ production began to exceed the live unamended control. CH_4_ production plateaued at ~0.91 mmol from ~300 d until ~500 d incubation, then increased gradually thereafter to 1.20 ± 0.03 mmol by day ~1200 (Figure 1A). Unexpectedly, no significant CH_4_ production was observed from CNUL cultures amended with M-5I throughout the ~1600 d incubation (Figure 1A). CNRL MFT amended with mixture M-3I exhibited a much longer lag time (~660 d) before beginning to produce more CH_4_ than the live control. Thereafter, CH_4_ was produced at a maximum rate of 5.0 µmol day^−1^, approximately equivalent to that measured in the parallel CNUL cultures (4.5 µmol day^−1^), with cumulative CH_4_ reaching 1.61 ± 0.05 mmol by ~1200 d (Figure 1B). In comparison, CNRL MFT amended with mixture M-5I endured an even longer lag phase of ~1200 d, after which CH_4_ was produced at the fastest rate measured in the experiment (8.5 µmol day^−1^) and accumulated the greatest mass of CH_4_ (2.45 ± 0.25 mmol) by ~1600 d (Figure 1B).

**Figure 2:**
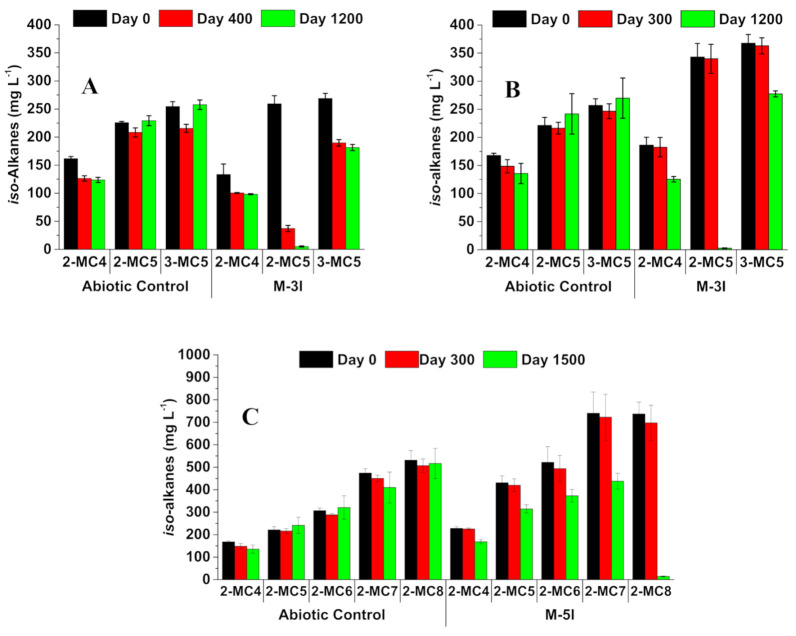
Concentrations of individual residual *iso*-alkanes in cultures sampled at day zero, during early CH_4_ production, and near the end of incubation. (**A**) CNUL MFT amended with the three *iso*-alkane mixture (CNUL M-3I); (**B**) CNRL MFT amended with the three *iso*-alkane mixture (CNRL M-3I); (**C**) CNRL MFT amended with the five *iso*-alkane mixture (CNRL M-5I). The bars represent the mean value of triplicate microcosms and error bars, where visible, represent one standard deviation.

Analysis of residual *iso*-alkanes in all abiotic control microcosms during incubation showed small differences in residual concentrations that generally were not statistically significant (Figure 2), indicating that abiotic loss was not a factor in determining biodegradation. Analysis of the live cultures amended with M-3I revealed similar patterns of biodegradation for both CNUL and CNRL: 2-MC_5_ was almost completely biodegraded whereas 2-MC_4_ and 3-MC_5_ were only partially depleted by ~1200 d incubation (Figure 2A,B). Approximately 30% of the initial 2-MC_4_ concentration (133 ± 18 and 186 ± 14 mg L^−1^ in CNUL and CNRL MFT, respectively) was depleted by ~1200 d, and ~25–33% of 3-MC_5_ (269 ± 8 and 368 ± 16 mg L^−1^ in CNUL and CNRL, respectively) was depleted (Figure 2A,B).

Because CNUL did not produce CH_4_ from the five-*iso*-alkane mixture (Figure 1A), we have no corresponding residual hydrocarbon data for these cultures, as chemical analysis was predicated on CH_4_ production. However, in CNRL cultures amended with M-5I, by ~1500 d incubation we observed preferential biodegradation of *iso*-alkanes in the sequence 2-MC_8_ > 2-MC_7_ = 2-MC_6_ = 2-MC_5_ > 2-MC_4_ (88%, 40%, 40%, 40%, and 28%, respectively, calculated for individual substrate depletion and corrected for abiotic controls; Table 1).

The masses of depleted *iso*-alkanes were translated into theoretical CH_4_ production using the modified Symons and Buswell stoichiometric Equations (1)–(6) to track the carbon flow from substrates to GHG via microbial metabolism (Table 1). For both CNUL and CNRL MFT amended with M-3I, the measured CH_4_ represented 46–55% of the theoretical maximum CH_4_ yield, whereas the CH_4_ measured from CNRL M-5I represented only 32% of the predicted CH_4_ yield (Table 1).

### 3.2. Microbial Communities Involved in Biodegradation of iso-Alkane Mixtures

The microbial community structures in CNUL and CNRL MFT cultures were monitored by pyrosequencing partial 16S rRNA genes. DNA sampling events for the M-3I cultures corresponded to the pre-amendment period (day 0), the early active methanogenic period (~300 d for CNUL and ~700 d for CNRL), and at the end of incubation after methanogenesis had ceased or slowed (~1600 d). In contrast, the CNRL cultures incubated with M-5I, the only suite to produce CH_4_ from M-5I, was sampled at day 0 and at the end of incubation because of the extremely long lag time: the culture was still producing methane (Figure 1) and likely still actively degrading *iso*-alkanes and/or pathway intermediates when sampled for DNA at 1600 d, thus resembling a mid- or late-active degradation phase community rather than a substrate-depleted community.

**Figure 3:**
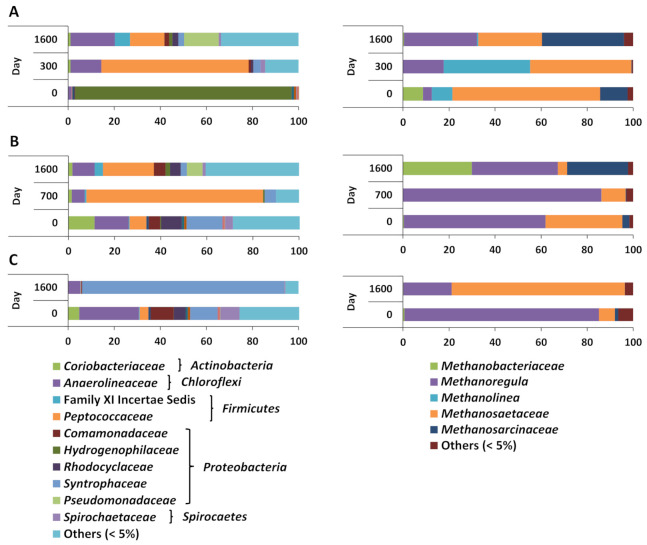
Bacterial (**left**) and archaeal (**right**) community compositions determined using 16S rRNA gene pyrosequencing before and during incubation of MFT amended with iso-alkane mixtures. (**A**) CNUL M-3I; (**B**) CNRL M-3I; (**C**) CNRL M-5I. The results represent pooled amplicons of triplicate cultures. Quality-controlled pyrosequences were clustered at ≤5% distance and expressed as a percentage of total archaeal or bacterial reads. “Others < 5%” is the sum of all taxa individually abundant at <5% of the total archaeal or bacterial reads in a given sample. Detailed results including the number of taxa grouped under “Others < 5%” are shown in Supplementary Tables S1 and S2.

Unsurprisingly, the two MFT sources had different microbial community compositions at day 0. Archaeal reads constituted only 22% of the total quality-controlled prokaryotic reads in CNUL MFT but two- to four-fold more reads (47–82%) in CNRL MFT (Supplementary Figure S1). Bacterial OTUs in CNUL MFT at day 0 were completely dominated by *Thiobacillus* OTUs in the family *Hydrogenophilaceae* (94%) with a low proportion of rare OTUs, whereas the initial CNRL MFT community was quite diverse, with ~40–50% of the bacterial reads represented by “rare” OTUs (<5% abundance across all microcosms) and the remainder of higher-abundance OTUs relatively evenly distributed among seven families (Figure 3 and Supplementary Table S1). The archaeal sequences in CNUL at day 0 were dominated by *Methanosaeta* (64%) in the Methanosarcinales, and those in CNRL by *Methanoregula* (62–85%) in the Methanomicrobiales (Figure 3 and Supplementary Table S2). These two genera are considered to be primarily acetoclastic and hydrogenotrophic, respectively, suggesting that the two MFT samples initially were acclimated to different dominant methanogenic activities prior to amendment with the *iso*-alkane mixtures.

During active metabolism of M-3I, the prokaryotic communities shifted to greater proportions of archaeal sequences relative to bacterial sequences, rising to >90% of total reads in both CNUL and CNRL (at 300 and 700 d, respectively) and remaining dominant until the end of incubation at 1600 d (Supplementary Figure S1). Within the reduced fraction of bacterial reads, the bacterial sequences in both CNUL and CNRL degrading M-3I became greatly enriched in OTUs affiliated with *Peptococcaceae* (61% and 77%, respectively; Figure 3 and Supplementary Table S1), suggesting a key role for these clostridial members in degrading low-molecular weight *iso*-alkanes. Overall bacterial diversity changed differently in CNUL and CNRL during active M-3I biodegradation: by 300 d CNUL had become more diverse as the proportion of *Hydrogenophilaceae* plummeted from 94% to undetectable levels and other OTUs including *Anaerolineaceae* and rare OTUs increased in abundance (12% and 23%, respectively) (Figure 3 and Supplementary Table S1). By the end of incubation (1600 d), CNUL incubated with M-3I harbored an even greater proportion of rare OTUs (43% of all bacterial reads) and a broader representation of families than in the original MFT. In contrast, CNRL incubated with M-3I experienced a transient decrease (from 48% to 14%) in rare bacterial OTUs during active methanogenesis (day 700) while *Peptococcaceae* dominated, then rebounded to the same proportion of rare OTUs (50%) by the end of incubation, suggesting that biodegradation was complete by 1600 d (Figure 3 and Supplementary Table S1). Interestingly, although the CNUL and CNRL communities were very different before incubation, their structures were quite similar by 1600 d.

The archaeal communities in all CNUL and CNRL cultures were overwhelmingly dominated by sequences affiliated with acetoclastic and hydrogenotrophic methanogens, but the taxonomic structures differed. In CNUL microcosms incubated with M-3I, the archaeal community that initially was dominated by acetoclastic *Methanosaeta* sequences shifted during active methanogenesis to include more sequences affiliated with hydrogenotrophic *Methanolinea*, which transiently increased in proportion at the expense of *Methanosaeta* (by 300 d). By the end of incubation (1600 d), *Methanolinea* had been replaced by *Methanosarcina* so that three genera in two different orders were approximately equal in proportion (Figure 3 and Supplementary Table S2) and represented a mix of acetoclastic and hydrogenotrophic genera. In contrast, CNRL incubated with M-3I harbored increased proportions of hydrogenotrophic *Methanoregula* and decreased acetoclastic *Methanosaeta* during active biodegradation (700 d). By the end of incubation, *Methanosarcina* (an acetoclastic genus) had emerged so that, as with CNUL, three genera were co-dominant; however, *Methanobacterium* (a hydrogenotrophic genus) had replaced *Methanosaeta* in the CNRL trio. That is, unlike the bacterial communities that converged on similar taxonomic structures after degrading M-3I, the archaeal communities in CNUL and CNRL still exhibited taxonomic differences by 1600 d.

The CNRL cultures incubated with M-5I developed bacterial and archaeal community structures distinct from the CNRL M-3I cultures. Strikingly, *Smithella*-related sequences dominated the bacterial reads, increasing from 9% initially to 86% by 1600 d (which we consider to represent mid- to late-active metabolism) and displacing the detectable rare biosphere sequences that decreased from 40% initially (0 d) to 8% of all reads by 1600 d. That is, the rare sequences had not rebounded as they did in the M-3I communities, again suggesting that biodegradation was still ongoing in the M-5I culture. Interestingly, the *Peptococcaceae*-related sequences that characterized the mid-phase communities degrading M-3I were not present in significant proportions in the M-5I culture at either sample point. By 1600 d, the archaeal community in CNRL incubated with M-5I differed from the original CNRL community and from both the M-3I communities, with *Methanoregula* (initially at 85%) being largely displaced by *Methanosaeta* (76%) by 1600 d (Figure 3 and Supplementary Table S2).

## 4. Discussion

Methanogenesis has both beneficial and detrimental potential for managing oil sands tailings ponds and end-pit lakes (a reclamation strategy for the tailings ponds [Kuznetsov et al., in preparation]). Possible benefits for tailings pond management include methanogenic acceleration of geochemical processes that affect settling of colloidal MFT clays and enhance recovery of porewater for re-use [26,27,36,37]. Conversely, the negative impacts on global warming of tailings ponds GHG emissions and the potential deleterious effects of microbial gas production on the long-term efficacy of end-pit lakes are concerning [38]. Therefore, understanding methanogenic metabolism of residual hydrocarbons in tailings is important for devising strategies for mitigating GHG emissions and ensuring sustainability of oil sands end-pit lakes where methanogenesis can affect both emissions and the quality of cap water. *n*-Alkanes and monoaromatics present in oil sands extraction solvents biodegrade relatively rapidly and completely under methanogenic conditions [20,21,24] but it is also important to know the fate of the more recalcitrant components of the solvents, such as *iso*-alkanes, after the more susceptible hydrocarbons have been depleted. Therefore, in the current study, we examined the biodegradability of selected *iso*-alkanes comprising part of the solvents that enter two different operators’ oil sands tailings ponds. The components of the M-3I mixture reflect the composition of the paraffinic solvent used by CNUL [8]. The M-5I mixture included two of the *iso*-alkanes in M-3I (2-MC_4_ and 2-MC_5_) that are also present in CNRL naphtha, plus three additional components (2-MC_6_, 2-MC_7_, and 2-MC_8_) selected to represent the higher molecular weight *iso*-alkanes detected in naphtha [25]. Furthermore, the specific isomers in M-5I were chosen with the methyl group in the same position as the two smaller *iso*-alkanes so as to detect any chain-length biodegradation preferences in the substrate series without introducing isomeric differences (i.e., 2-MC_4–8_).

Methanogenic biodegradation of the M-3I mixture began earlier in CNUL MFT than CNRL MFT. The simplest explanation is that the CNUL community was already adapted to the *iso*-alkanes comprising M-3I because they are major components of the paraffinic solvent in CNUL ponds [11]. Recent reports [11,21,25] have shown similar observations, where a longer lag phase was observed when methanogenic MFT from different oil sands operators were cross-fed with hydrocarbon mixtures that were ‘exotic’ to the respective tailings ponds. The results support the general concept that acclimation is an important step before the indigenous methanogenic microbial communities in MFT begin to oxidize exotic substrates. The rate of CH_4_ production from M-3I was similar for both CNUL and CNRL cultures, as was the pattern of substrate depletion: 2-MC_5_ was completely depleted whereas 2-MC_4_ and 3-MC_5_ were only partially removed, and their depletion apparently ceased once 2-MC_5_ was gone. This suggests co-metabolism of 2-MC_4_ and 3-MC_5_ in the presence of 2-MC_5_, consistent with the pattern observed with Syncrude MFT [8,9,39]. Amending the two MFT samples with the M-5I suite of *iso*-alkanes yielded different results. First, after a very lengthy lag time CNRL produced CH_4_ at a rate ~1.7-fold greater than that calculated for M-3I, consistent with the longer carbon chain length of the substrates. In contrast, even after ~1600 d incubation CNUL MFT failed to produce CH_4_ from the broader range of *iso*-alkanes, despite two of the M-5I components being successfully degraded when added in M-3I. This result was unexpected because CNUL previously had been shown to degrade naphtha-range *n*- and *iso*-alkanes via methanogenesis [25]. A possible explanation is that the total concentration of *iso*-alkanes introduced via the M-5I mixture (~2.6 mmol; Table 1) versus M-3I (~1 mmol) might have proven toxic to CNUL community, or individual *iso*-alkanes might have been inhibitory. Incidentally, inhibition might also explain the 2-fold longer lag period exhibited by CNRL amended with M-5I compared with M-3I. Perhaps extending incubation beyond 1600 d would have allowed CNUL to overcome a lengthy lag period and eventually produce CH_4_ from M-5I; notably, lag phases of up to 1800 d (~5 years) have been reported for methanogenic degradation of similar *iso*-alkanes by Syncrude MFT [8]. Another possibility is that CNUL lacked the requisite bacterial partners for degradation of mid-chain length *iso*-alkanes, discussed below.

The second observation related to M-5I is that CNRL MFT exhibited a pattern of preferential degradation of the suite of 2-methyl-alkanes in order of decreasing carbon chain length from 2-MC_8_ to 2-MC_4_. This pattern is analogous to that observed for biodegradation of a C_10_–C_5_ *n*-alkane mixture by CNRL [21] and also degradation of C_10_–C_6_ *n*-alkanes in naphtha by Syncrude [20], but opposite to the trend observed with CNUL degrading a mixture of C_5_–C_10_ *n*-alkanes [21]. The preferential biodegradation pattern in CNRL MFT amended with M-5I might be attributed to the lower toxicity of longer-chain *iso*-alkanes compared to the relatively more water-soluble shorter-chain *iso*-alkanes [40]. The presence of other hydrocarbons (e.g., mixtures of pure alkanes versus whole naphtha or paraffinic solvent) may also affect the order of biodegradation.

Overall, methanogenic oxidation of *iso*-alkanes in MFT was inefficient since the measured methane was only 32 to 55% of the theoretical yields based on the stoichiometric conversion of the *iso*-alkanes. Similar stoichiometric values (23–44%; [10,39]) have been reported in recent studies examining methanogenic degradation of *iso*-alkanes in MFT and in production water from a high-temperature petroleum reservoir [41]. In contrast to the current *iso*-alkane study, biodegradation of *n*-alkanes under methanogenic conditions reached ~60–80% of theoretical CH_4_ production [20,21,42]. Possible reasons for the low CH_4_ yield in the current study could be: (1) co-metabolism of 2-MC_4_ and 3-MC_5_ during oxidation of 2-MC_5_ in CNUL and CNRL MFT amended with M-3I, and of 2-MC4 (at least) in M-5I. This phenomenon of incomplete oxidation may lead to accumulation of dead-end products that decrease CH_4_ efficiency, and/or (2) high incorporation of the substrate carbon into biomass to maintain the diverse microbial communities in MFT [39,41].

To identify the key microbial taxa involved in *iso*-alkane biodegradation, we performed microbial community analysis on culture samples withdrawn at day 0, during active CH_4_ production and/or at the end of incubation. The initial bacterial community in CNUL MFT was dominated by *Thiobacillus* OTUs in the family *Hydrogenophilaceae*, as reported in previous studies [21]. The genus includes facultatively anaerobic species that can oxidize ferrous iron, iron sulfide, and pyrite using nitrate as electron acceptor [43,44]; these iron minerals are found in MFT [27] although it is unclear whether this activity supports the abundance of *Thiobacillus* in stored CNUL MFT. During methanogenic biodegradation of the short-chain *iso*-alkanes in M-3I, sequences related to *Peptococcaceae* were enriched in both CNUL and CNRL MFT, implicating members of this family in the primary degradation of 2-MC_5_ and likely in co-metabolism of 2-MC_4_ and 3-MC_5_ under methanogenic conditions. Similar observations of *Peptococcaceae* enrichment during methanogenic biodegradation of *iso*-alkanes have been reported in recent studies [8,9,10,39,41], and some *Peptococcaceae* genomes have been shown to carry the appropriate genes for activation of alkanes via fumarate addition pathway [45,46].

Interestingly, in the CNRL M-5I culture the predominant OTUs were affiliated with *Smithella* rather than *Peptococcaceae.* This is consistent with several previous studies of different MFT samples in which *Peptococcaceae* OTUs predominated during short-chain alkane (≤C_6_) incubation but *Smithella* (and/or the closely related genus *Syntrophus*) was enriched with mid-chain length *n*- and *iso*-alkanes (>C_6_) [9,21,25]. Previous genomic [47] and metabolic [48] investigations showed that *Smithella* spp. might degrade alkanes via a fumarate addition pathway, and genes for this activity related to *Smithella* genes were detected during biodegradation of a mixture of 2-, 3- and 4-methyloctane in cultures derived from production water from a high-temperature petroleum reservoir [41]. The presence of *Smithella* in the initial MFT samples then might explain why CNUL did not produce CH_4_ from M-5I. In the current study, *Smithella*-affiliated sequences were below detection levels in CNUL at day 0 and reached a maximum of 1% abundance, whereas CNRL MFT harbored a substantial proportion (~9%) of sequences affiliated with *Smithella* even at day 0 that became highly enriched (86%) during active biodegradation of M-5I concomitant with CH_4_ production. Therefore, the cumulative conclusion from the current study and our previous studies with CNRL, CNUL, and Syncrude MFT samples [21,22,25,42] is that *Smithella* is the primary degrader for longer-chain (>C_6_) *n*- and *iso*-alkanes under methanogenic conditions and *Peptococcaceae* for short-chain *iso*-alkanes.

Sequences related to *Anaerolineaceae* were also detected in all cultures during *iso*-alkane degradation, suggesting the possibility that they are primary degraders [11,49]. However, since *Anaerolineaceae* is only present at moderate abundance (3–12% during active methanogenesis), and in fact decreased in abundance in CNRL during incubation, it is more likely that *Anaerolineaceae* are partners for the primary degraders, serving as autotrophic carbon fixers or H_2_ scavengers [39].

The archaeal communities in CNUL and CNRL M-3I were dominated by hydrogenotrophic methanogens during active degradation. Similar observations have also been reported in other methanogenic *iso*-alkane-degrading primary cultures derived from Syncrude MFT [8,10], highlighting the importance of hydrogenotrophic methanogenesis in oil sands tailings ponds. The inefficiency of methanogenic *iso*-alkane metabolism discussed above may have fostered the shift from acetoclastic to hydrogenotrophic metabolism during utilization of the short-chain M-3I mixtures. In contrast, the archaeal community in CNRL M-5I that initially was dominated by hydrogenotrophic archaeal OTUs (85%), became highly enriched in acetoclastic OTUs (75%) during mineralization of the longer-chain *iso*-alkanes (>C_6_), similar to previous reports of MFT cultures grown on *n*-alkanes [21,22] and long-chain *iso*-alkanes/cycloalkanes [9]. It is possible that methanogenic oxidation of long-chain *iso*-alkanes, despite the apparent inefficiency, was more favorable for acetoclastic metabolism, similar to methanogenic *n*-alkane degradation.

In summary, the current study demonstrates that, similar to Syncrude MFT [8,10,39], microbial communities indigenous to CNUL and CNRL tailings ponds are capable of degrading *iso*-alkanes. Our results confirm that co-metabolism of short-chain *iso*-alkanes might also be an important activity in MFT. The current study also demonstrates that the indigenous microbial community in CNRL MFT preferentially oxidizes *iso*-alkanes in the order of decreasing molecular weights and is capable of metabolizing a wide range of *iso*-alkanes. However, the CNUL MFT that is acclimated to short-chain *n*- and *iso*-alkanes was incapable of degrading mid-chain length *iso*-alkanes during ~1600 d incubation, emphasizing that each oil sands tailings pond is unique [19]. These results are important for understanding the *iso*-alkane degradation process, which could improve the current oil sands tailings greenhouse gas emission model [15] and impact future decisions regarding oil sands operations remediation and management; for example, anticipating lengthy lag times for onset of methanogenesis in newly established tailings ponds, and predicting prolonged methane production from end-pit lakes where *iso*-alkanes are a significant component of the residual hydrocarbons.

## Figures and Tables

**Table 1:** Predicted and measured methane production in mature fine tailings with the degradation of *iso*-alkanes after incubation.

	Substrate	Incubation Time (d)	Substrate Added at Day 0 (mmol)	Substrate Consumed (mmol) ǂ	Theoretical Methane Yield * (mmol)	Total Predicted Methane Yield * (mmol)	Total Measured Methane Yield (mmol)	Percent of Theoretical Production (%)
CNUL M-3I	2-MC4	392	0.26 (±0.02)	0.11 (±0.02)	0.46 (±0.09)	1.99 (±0.15)	0.91 (±0.04)	45.7
	2-MC5		0.30 (±0.20)	0.22 (±0.01)	1.05 (±0.05)			
	3-MC5		0.31 (±0.01)	0.10 (±0.00)	0.48 (±0.02)			
CNRL M-3I	2-MC4	1172	0.26 (±0.02)	0.08 (±0.01)	0.34 (±0.04)	2.93 (±0.23)	1.61 (±0.05)	55.0
	2-MC5		0.40 (±0.03)	0.39 (±0.03)	1.85 (±0.13)			
	3-MC5		0.43 (±0.02)	0.16 (±0.01)	0.75 (±0.06)			
CNRL M-5I	2-MC4	1459	0.32 (±0.01)	0.09 (±0.00)	0.30 (±0.00)	7.57 (±0.86)	2.43 (±0.25)	32.2
	2-MC5		0.50 (±0.03)	0.20 (±0.02)	0.79 (±0.09)			
	2-MC6		0.53 (±0.05)	0.21 (±0.04)	0.94 (±0.24)			
	2-MC7		0.64 (±0.06)	0.26 (±0.05)	1.45 (±0.28)			
	2-MC8		0.57 (±0.03)	0.50 (±0.03)	3.46 (±0.24)			

ǂ Calculation was made based on the difference of measured day 0 concentrations, taking into account abiotic losses, and residual alkane concentrations at incubation time indicated. * Calculation based on Equations (1)–(6) using masses (GC quantitation) of the consumed *iso*-alkanes. Values represent the mean from analysis of triplicate microcosms (±1 standard deviation).

## Data Availability

Data available in a publicly accessible repository that does not issue DOIs. Publicly available datasets were analyzed in this study. The data were submitted to NCBI Sequence Read Archive under SRA number SRP052814.

## Supplementary Materials

The following are available online at https://www.mdpi.com/article/10.3390/microorganisms9081569/s1, Figure S1: Archaeal and bacterial reads expressed as a proportion of total quality-controlled prokaryotic reads, based on analysis of partial 16S rRNA gene pyrosequences before and during incubation of MFT with *iso*-alkanes mixtures. Table S1: Relative abundance of bacterial 16S rRNA gene pyrosequences, grouped at class level, in CNUL and CNRL MFT incubated with mixtures of either three or five *iso*-alkanes (M-3I or M-5I) under methanogenic conditions. Table S2: Relative abundance of archaeal 16S rRNA gene pyrosequences, grouped at class level, in CNUL and CNRL MFT incubated with mixtures of either three or five *iso*-alkanes (M-3I or M-5I) under methanogenic conditions.
