# Supplementary material for: Methanogenic Biodegradation of iso-Alkanes by Indigenous Microbes from Two Different Oil Sands Tailings Ponds
Source: Microorganisms. 2021 Jul 23;9(8):1569. doi: 10.3390/microorganisms9081569 (PMC8400375; doi:10.3390/microorganisms9081569)
Supplement: Supplementary file 1 [file microorganisms-09-01569-s001.zip › microorganisms-1291341-supplementary.pdf]

microorganisms

SUPPLEMENTARY MATERIALS FOR

Methanogenic Biodegradation of *iso*-Alkanes by Indigenous Microbes from Two Different Oil Sands Tailings Ponds

Mohd Faidz Mohamad Shahimin [1,†], Julia M. Foght [2] and Tariq Siddique [1,*]

[1] Department of Renewable Resources, University of Alberta, Edmonton, AB T6G 2G7, Canada
[2] Department of Biological Sciences, University of Alberta, Edmonton, AB, T6G 2E9, Canada
* Correspondence: tariq.siddique@ualberta.ca
† Current Address: Department of Chemical Engineering Technology, Universiti Malaysia Perlis, Padang Besar, 02100 Perlis, Malaysia.

The supplementary materials contain 1 figure (Figure S1) and 2 tables (Table S1 and S2).

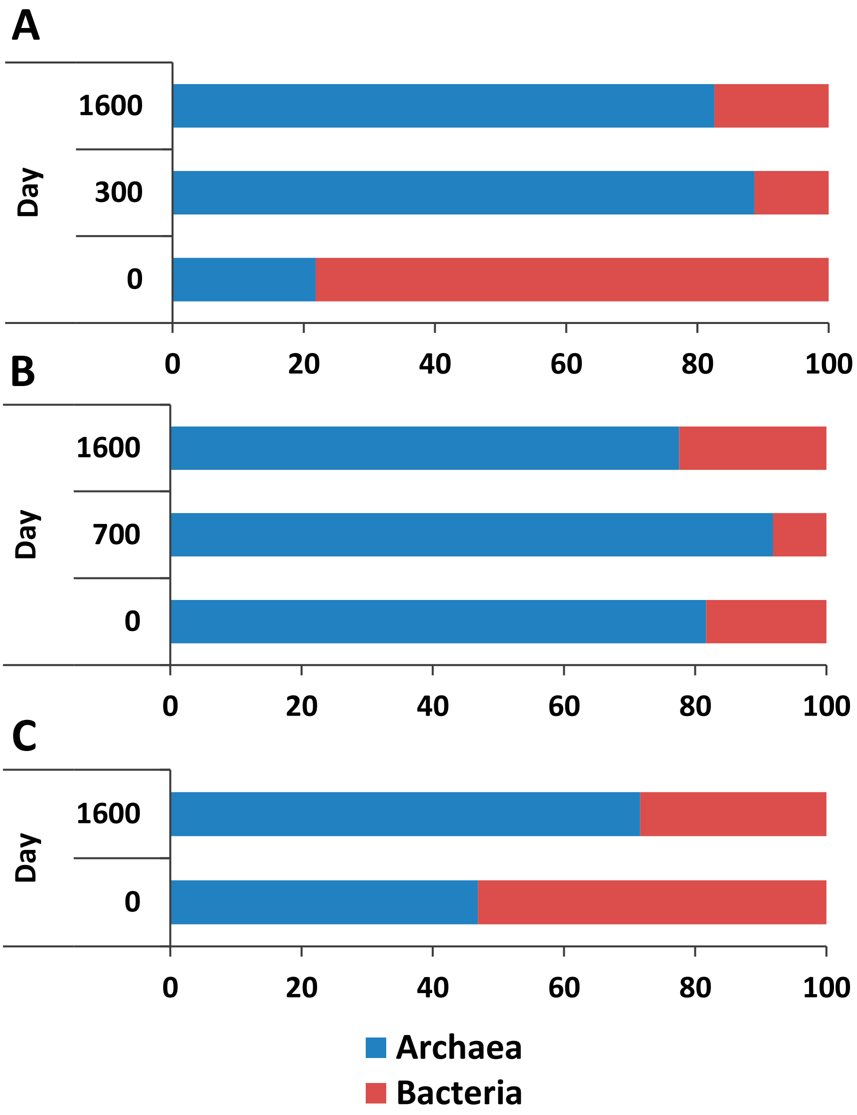

Figure S1. Archaeal and bacterial reads expressed as a proportion of total quality-controlled prokaryotic reads, based on analysis of partial 16S rRNA gene pyrosequences before and during incubation of MFT with *iso*-alkanes mixtures. (**A**) CNUL MFT amended with M-3I; (**B**) CNRL MFT amended with M-3I; and (**C**) CNRL MFT amended with M-5I. The results represent pooled amplicons of triplicate cultures.

Table S1. Relative abundance of bacterial 16S rRNA gene pyrosequences, grouped at class level, in CNUL and CNRL MFT incubated with mixtures of either three or five *iso*-alkanes (M-3I or M-5I) under methanogenic conditions. Analyses were performed on MFT before degradation (day 0) and after incubation times indicated in days. Genus assignations are shown where confident. "Others <5%" is the sum of all taxa individually abundant at <5% of the total bacterial reads in a given sample.

Nomenclature	CNUL			CNRL				
	M-3I			M-3I			M-5I	
	Day							
	0	300	1600	0	700	1600	0	1600
Actinobacteria								
Coriobacteriales;Coriobacterineae;Coriobacteriaceae	0.1	1.0	1.0	11.3	1.4	1.7	4.9	0.2
Anaerolineae								
Anaerolineales;Anaerolineaceae;Leptolinea	0.6	5.1	3.5	3.6	2.8	1.2	7.2	1.6
*Anaerolineales;Anaerolineaceae;*uncultured	0.4	7.2	12.2	10.3	2.8	4.0	13.8	2.7
Clostridia								
*Clostridiales;*Family XI Incertae Sedis	0.0	0.0	6.2	0.4	0.7	2.9	0.3	0.1
Clostridiales;Peptococcaceae	0.2	60.8	14.1	3.6	76.6	21.0	2.6	0.0
Betaproteobacteria								
Burkholderiales;Comamonadaceae	0.1	0.0	1.8	0.2	0.0	4.7	8.9	0.3
Hydrogenophilales;Hydrogenophilaceae;Thiobacillus	93.8	0.0	1.3	0.3	0.7	1.8	0.1	0.0
Rhodocyclales;Rhodocyclaceae								
*Rhodocyclales;Rhodocyclaceae;*uncultured	0.0	0.0	2.3	8.9	0.0	4.0	4.5	0.1
Deltaproteobacteria								
Syntrophobacterales;Syntrophaceae;Smithella	0.0	1.0	0.5	9.7	1.4	1.8	8.5	86.1
Gammaproteobacteria								
Pseudomonadales;Pseudomonadaceae;Pseudomonas	0.6	0.0	12.9	0.1	0.0	5.6	0.2	0.1
Spirochaetes								
*Spirochaetales;Spirochaetaceae;*uncultured	0.0	2.1	1.0	3.4	0.0	1.3	8.0	0.4
**Others (< 5%)**	4.1	22.7	43.3	48.2	13.5	50.1	40.9	8.3

Table S2. Relative abundance of archaeal 16S rRNA gene pyrosequences, grouped at class level, in CNUL and CNRL MFT incubated with mixtures of either three or five *iso*-alkanes (M-3I or M-5I) under methanogenic conditions. Analyses were performed on MFT before degradation (day 0) and after incubation times indicated. Genus assignations are shown where confident. "Others <5%" is the sum of all taxa individually abundant at <5% of the total archaeal reads in a given sample.

Nomenclature	CNUL			CNRL				
	M-3I			M-3I			M-5I	
	Day							
	0	300	1600	0	700	1600	0	1600
Methanobacteriales								
Methanobacteriaceae;Methanobacterium	8.5	0.0	0.4	0.4	0.0	29.7	0.6	0.2
Methanomicrobiales								
Methanoregula	3.9	17.6	31.7	61.5	86.1	37.3	84.5	21.0
Methanolinea	8.9	37.6	0.6	0.0	0.1	0.0	0.0	0.0
Methanosarcinales								
Methanosaetaceae;Methanosaeta	64.1	43.9	27.7	33.3	10.5	4.0	6.9	75.2
Methanosarcinaceae;Methanosarcina	11.2	0.4	35.4	0.3	0.3	26.3	0.2	0.0
**Others (< 5%)**	3.3	0.4	4.2	4.4	3.0	2.7	7.9	3.6
